# Hypoxia/Ischemia-Induced Rod Microglia Phenotype in CA1 Hippocampal Slices

**DOI:** 10.3390/ijms23031422

**Published:** 2022-01-26

**Authors:** Daniele Lana, Elisabetta Gerace, Giada Magni, Francesca Cialdai, Monica Monici, Guido Mannaioni, Maria Grazia Giovannini

**Affiliations:** 1 Section of Clinical Pharmacology and Oncology, Department of Health Sciences, University of Florence, 50139 Florence, Italy; mariagrazia.giovannini@unifi.it; 2 Section of Pharmacology and Toxicology, Department of Neuroscience, Psychology, Drug Research and Child Health (NeuroFarBa), University of Florence, 50139 Florence, Italy; elisabetta.gerace@unifi.it (E.G.); guido.mannaioni@unifi.it (G.M.); 3 Institute of Applied Physics “Nello Carrara”, National Research Council (IFAC-CNR), 50019 Sesto Fiorentino, Italy; g.magni@ifac.cnr.it; 4 ASA Research Division, ASA Campus Joint Laboratory, Department of Experimental and Clinical Biomedical Sciences “Mario Serio”, University of Florence, 50134 Florence, Italy; francesca.cialdai@unifi.it (F.C.); monica.monici@unifi.it (M.M.)

**Keywords:** oxygen glucose deprivation, immunofluorescence, phagocytosis, neurodegeneration, rod microglia train, confocal microscopy, CA3 hippocampus

## Abstract

The complexity of microglia phenotypes and their related functions compels the continuous study of microglia in diseases animal models. We demonstrated that oxygen-glucose deprivation (OGD) induced rapid, time- and space-dependent phenotypic microglia modifications in CA1 stratum pyramidalis (SP) and stratum radiatum (SR) of rat organotypic hippocampal slices as well as the degeneration of pyramidal neurons, especially in the outer layer of SP. Twenty-four h following OGD, many rod microglia formed trains of elongated cells spanning from the SR throughout the CA1, reaching the SP outer layer where they acquired a round-shaped amoeboid phagocytic head and phagocytosed most of the pyknotic, damaged neurons. NIR-laser treatment, known to preserve neuronal viability after OGD, prevented rod microglia formation. In CA3 SP, pyramidal neurons were less damaged, no rod microglia were found. Thirty-six h after OGD, neuronal damage was more pronounced in SP outer and inner layers of CA1, rod microglia cells were no longer detectable, and most microglia were amoeboid/phagocytic. Damaged neurons, more numerous 36 h after OGD, were phagocytosed by amoeboid microglia in both inner and outer layers of CA1. In response to OGD, microglia can acquire different morphofunctional phenotypes which depend on the time after the insult and on the subregion where microglia are located.

## 1. Introduction

Microglia, the resident immune cells of the central nervous system, represent 5–10% of the population of brain cells [1,2]. In physiological conditions, resting microglia have a small soma and fine, highly mobile, and ramified branches, which dynamically patrol the brain parenchyma [3]. Microglia activate rapidly and are first responders in many neurodegenerative insults such as ischemic brain injury [4] and become major actors of the neuroinflammatory response [5,6].

Microglia are plastic cells that undergo profound functional modifications in response to damaging stimuli. An oversimplified view recognizes two extremes of profound functional reprogramming in response to cytokines, chemokines and other soluble factors produced by damaged neurons, the classical pro-inflammatory and the anti-inflammatory phenotypes [7,8,9,10,11,12,13]. Indeed, factors released by damaged cells polarize microglia toward a M1 phenotype, driving secondary brain injury. However, microglia can also polarize toward a M2 phenotype, contributing to the suppression of the neuroinflammatory response. Between these extreme functional states, a plethora of possible intermediate states are recognized, among which the Rod microglia phenotype has recently come into focus, raising interest on its still unknown functions [14,15,16,17,18,19].

Microglia patrol the parenchyma, detecting and eliminating damaged neurons by phagocytosis [3,20,21,22,23] and maintaining a healthy environment. Since microglia projections are chemotactic sensors that extend towards injured cells in the “find-me” step of neuron phagocytosis [7], their impairment may weaken the neuroprotective activity of microglia. A decreased migration of microglia may hamper its phagocytic efficacy, favoring the accumulation of degenerating neurons and neuronal toxic debris [24].

In 1899, F. Nissl described rod cells in the general paralysis of the insane [25], and their existence was later confirmed by Alois Alzheimer (1904) and others. In 1922 Spielmeyer found rod microglia in the cerebral cortex of patients with neurological disorders associated with typhus infections and syphilis [26]. These cells, aligned with nerve cells, were depicted as “strung-out, long, slim glial cells with rod-like nuclei perpendicular to the cortical surface” [27].

Although known for over a century, rod microglia were long forgotten and “re-discovered” much later, in 2012, by Ziebell and colleagues [14] in a rat model of diffuse traumatic brain injury. It was shown that the elongated microglia cell bodies form trains adjacent to apical dendrites and perpendicular to the dural surface [14,15,28]. Since then, some work has been done to identify and characterize rod microglia in the injured brain.

Most of the reported data were obtained in the cortex, in models of traumatic brain injury [14,15,29]. In 2015 Bachstetter and coworkers demonstrated that trains of rod microglia are predominantly aligned end-to-end in the CA1 and CA2/3 hippocampus of patient with AD, dementia with Lewy bodies and hippocampal sclerosis in the aged brain [30]. In a rat model of global ischemia, rod microglia were found in the substratum radiatum 10 days after the insult [31].

The complexity of microglia phenotypes and their related functions compels the continuous study of microglia in animal models of diseases. Indeed, since studies on rod microglia are limited, very little is known on the physiological and/or pathological role of this microglia variant in health and disease, and particularly in brain ischemia.

Our experiments will hopefully lay the basis for the ambitious aim to understand the molecular pathways that drive cell–cell interactions, to intercept microglia activation and hopefully to re-educate these cells toward their physiological protective activity.

## 2. Results

Using organotypic hippocampal slices, we assessed whether oxygen glucose deprivation (OGD), an in vitro simil-ischemic insult that causes neurotoxicity [32], evokes activation and/or phenotypic changes of microglia. To this aim, we performed triple immunostaining and confocal microscopy to visualize resting microglia with anti-IBA1 antibody and its activation with anti-MHCII (OX6) antibody, respectively. Neuronal damage was assessed visualizing pyknotic or karyorrhectic neurons with anti-NeuN antibody. We also assessed the effect of a NIR-laser light treatment (7.42 J/cm^2^) applied one time immediately after OGD on all the parameters investigated. NIR-laser application on control slices did not provoke any damaging effect by itself, neither on neurons nor on microglia.

**Figure 1:**
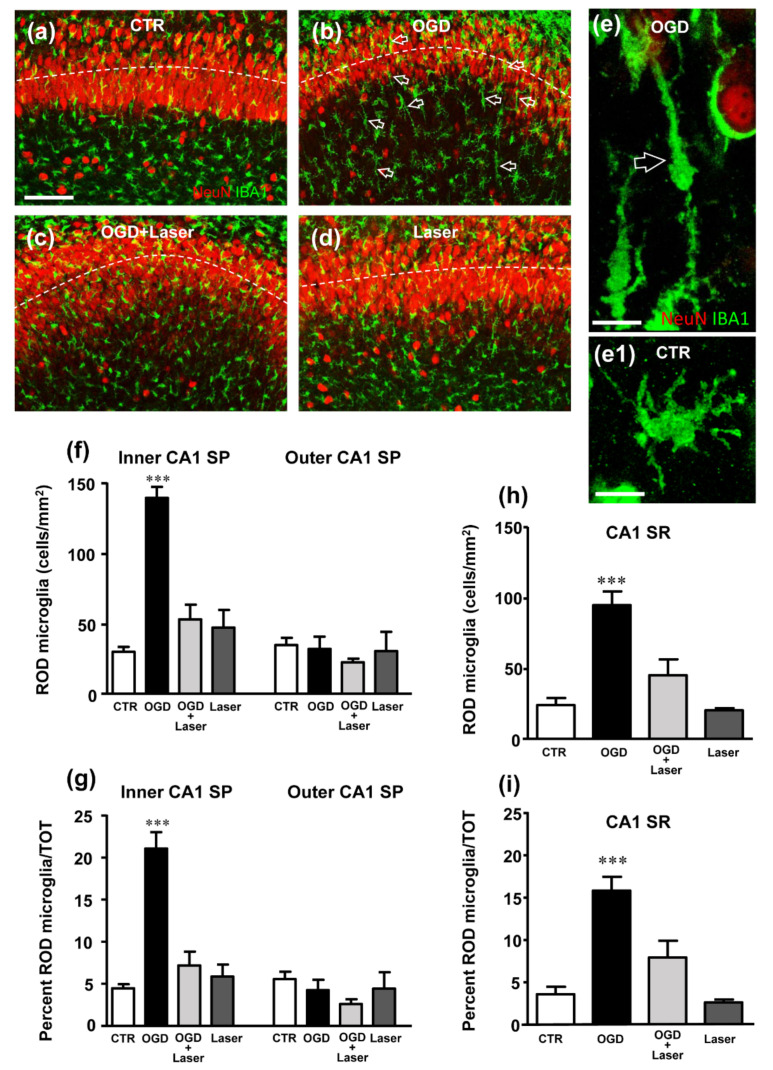
(**a**–**d**) Representative confocal images of NeuN+ neurons (red) and IBA1+ total microglia (green) in CA1 hippocampus of a CTR (**a**), an OGD (**b**), an OGD+Laser (**c**) and a Laser (**d**) organotypic hippocampal slices captured with a 20× objective. Images were obtained by stacking 3 consecutive confocal scans (z step 1.2 µm, total thickness 3.6 µm). Numerous rod microglia are present in CA1 SP and CA1 SR of OGD slices (open arrows). The dotted lines delineate the inner and outer CA1 SP layers. Scale bar: 50 µm. (**e**) Confocal 3D-rendering of an IBA1-positive rod microglia (green, open arrow) NeuN-positive neurons (red), captured with a 63× objective in an OGD organotypic hippocampal slice. The 3D-rendering was obtained stacking 15 consecutive confocal scans (z step 0.3 µm, total thickness 4.5 µm). Scale bar: 20 µm. (**e1**) Confocal 3D-rendering of an IBA1-positive (green) resting microglia captured with a 63× objective in a CTR organotypic hippocampal slice. The 3D-rendering is obtained stacking 12 consecutive confocal scans (z step 0.3 µm, total thickness 3.6 µm). Scale bar: 20 µm. (**f**) Quantitative analyses of rod microglia density (cells/mm^2^) in the inner and outer layers of CA1 SP. Statistical analysis: Inner CA1 SP: *** *p* < 0.001, OGD vs. all other experimental groups; Outer CA1 SP: no significant differences were found among the 4 experimental groups. (**g**) Quantitative analyses of the percent of rod microglia on total microglia in inner and outer layers of CA1 SP. Statistical analysis: Inner CA1 SP: *** *p* < 0.001, OGD vs. all experimental groups; Outer CA1 SP: no significant differences were found among the 4 experimental groups; (**h**) Quantitative analyses of rod microglia density (cells/mm^2^) in CA1 SR. Statistical analysis: *** *p* < 0.001 OGD vs. all experimental groups. (**i**) Quantitative analyses of the percent of rod microglia on total microglia in CA1 SR. Statistical analysis: *** *p* < 0.001 OGD vs. all experimental groups. In the bar graphs, each bar represents the mean ± SEM of 5–7 independent slices. All statistical analyses were performed by One-way ANOVA followed by Newman-Keuls post hoc test.

The immunostaining of resting microglia with IBA1 antibody showed that 24 h after 30 min of OGD, the morphology of many microglia cells, mainly located in both the Stratum Radiatum (SR) and the inner layer of the CA1 SP (see below) had acquired an elongated, rod-like morphology, with their longer axis perpendicular to the CA1 SP. These rod microglia were localized mainly in the SR and in the inner half of the SP (Figure 1b,e, open arrows). The rod microglia (Figure 1e) appeared to have an elongated cell body (Figure 1e, open arrow) and had lost most of their thinner branches. The length of the microglia cells varied between 40 µm to more than 100 µm, similar to the one shown in Figure 1e, which was around 80 µm long. As a comparison, a typical resting microglia cell is shown in Figure 1e1.

We carried out the quantitative analysis of rod microglia in control, OGD, OGD+Laser and Laser-treated slices, considering only microglia cells longer than 40 µm. As shown (Figure 1f,i), it is possible to appreciate that the density of rod microglia is increased in the inner layer of CA1 SP in OGD-treated slices (+355% vs. controls, *** *p* < 0.001 vs. all other groups, Figure 1f) and in SR (+290% vs. controls, **p < 0.01 vs. all other groups, Figure 1g), while no changes were observed in the outer layer of CA1 SP. In addition, rod microglia, expressed as percent of total microglia, increased significantly in the inner layer of CA1 SP (+370% vs. controls, *** *p* < 0.001 vs. all other groups, Figure 1h) and in SR (+240% vs. controls, *** *p* < 0.001 vs. all other groups Figure 1i), but not in the outer layer of CA1 SP. Although NIR-Laser application to control slices had no effect per se, it completely blocked the appearance of rod microglia in the inner SP and SR of OGD-treated slices (Figure 1f–i).

**Figure 2:**
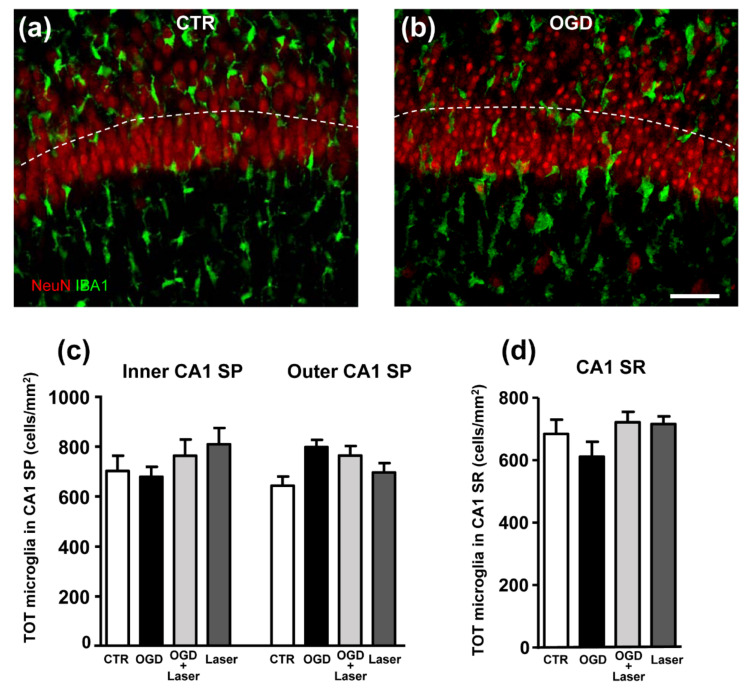
(**a**,**b**) Representative confocal images of NeuN-positive neurons (red), and IBA1-positive total microglia (green) in CA1 of a CTR (**a**), and an OGD (**b**) organotypic hippocampal slice captured with a 40× objective. The images were obtained stacking 5 consecutive confocal scans (z step 0.5 µm, total thickness 2.5 µm). The dotted lines delineate the inner and outer CA1 SP layers. Scale bar: 20 µm. (**c**) Quantitative analyses of total microglia density (cells/mm^2^) in the inner and outer layers of CA1 SP. Statistical analysis: n.s. (**d**) Quantitative analyses of total microglia density (cells/mm^2^) in CA1 SR. Statistical analysis: n.s. In the bar graphs, each bar represents the mean ± SEM of 6–7 independent slices. All statistical analyses were performed by One-way ANOVA followed by Newman-Keuls post hoc test.

We verified whether an OGD insult might modify the density of microglia in CA1 SP or SR. As apparent in the quantitative analysis of total microglia in the inner and outer SP layers and in the SR of control, OGD, OGD+Laser and Laser-treated slices, we demonstrated that there were no significant differences of microglia density in any of these groups (Figure 2). These data demonstrated that the increase in rod microglia in the outer layer of SP was due to morphological modifications of existing cells and not to increase in the microglia cell number (Figure 2c,d), thus confirming previous findings that showed that rod microglia originate by morphofunctional modifications from resident CNS microglia.

**Figure 3:**
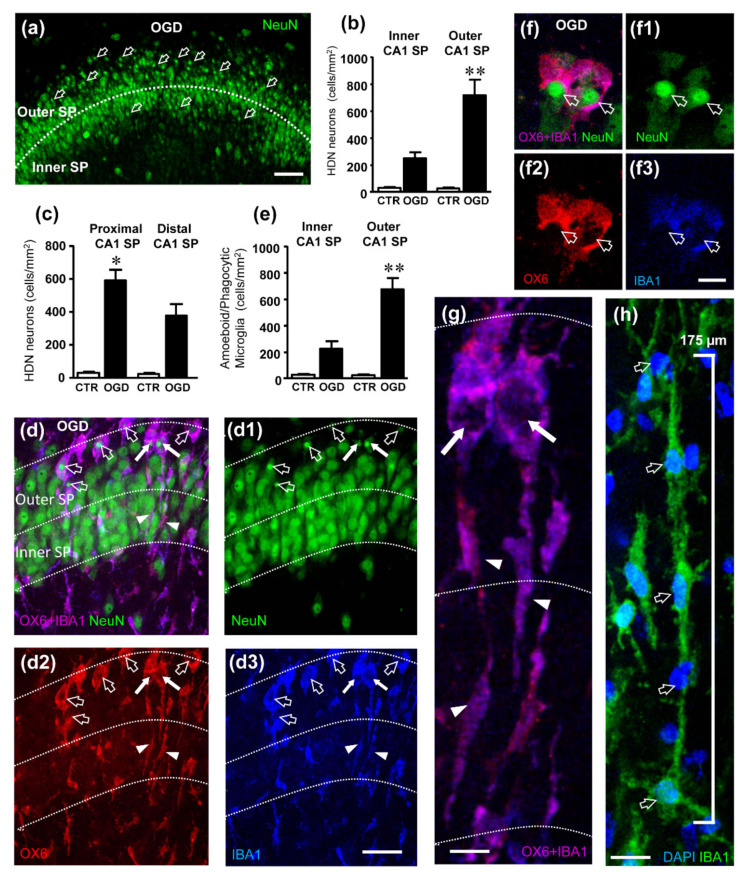
(**a**) Representative confocal images of NeuN-positive neurons (green) in CA1 of an OGD organotypic hippocampal slice. The image was obtained from a single confocal scan captured with a 20× objective, total thickness 1.2 µm. The dotted line delineates the inner and outer CA1 SP layers. Numerous HDN neurons (open arrows) are located mainly in the outer layer of CA1 SP. Scale bar: 50 µm. (**b**) Quantitative analyses of HDN neurons density (cells/mm^2^) in the inner and outer CA1 SP. Statistical analysis: ** *p* < 0.01, Outer CA1 SP OGD vs. Inner CA1 SP OGD. (**c**) Quantitative analyses of HDN neurons density (cells/mm^2^) in the proximal and distal CA1 SP. Statistical analysis: * *p* < 0.05, Distal CA1 SP OGD vs. Proximal CA1 SP OGD. (**d**–**d3**) Representative confocal images of NeuN-positive neurons (green, (**d1**)), OX6-positive active microglia (red, (**d2**)) and IBA1-positive total microglia (blue, (**d3**)) in CA1 of an OGD organotypic hippocampal slice captured with a 63× objective. The merge of the three immunofluorescent staining is shown in (**d**) in which the pink-purple color is indicative of active (OX6-positive) microglia cells (IBA1-positive). The images were obtained stacking 10 consecutive confocal scans (z step 0.3 µm, total thickness 3 µm). Numerous active microglia cell bodies phagocytosing HDN neurons are present in the outer CA1 SP (open arrows). The dotted lines delineate the inner and outer CA1 SP. Scale bar: 25 µm. (**e**) Quantitative analyses of the density of phagocytic microglia (cells/mm^2^) in the inner and outer layers of CA1 SP. Statistical analysis: ** *p* < 0.01, Outer CA1 SP OGD vs. Inner CA1 SP OGD. In the bar graphs, each bar represents the mean ± SEM of 7–9 independent slices. All statistical analyses were performed by Student’s paired *t* test. (**f**–**f3**) Representative confocal images of NeuN-positive neurons (green, (**f1**)), OX6-positive active microglia (red, (**f2**)) and IBA1-positive total microglia (blue, (**f3**)) in CA1 of an OGD organotypic hippocampal slice captured with a 63× objective. The merge of the three immunofluorescent staining is shown in (**f**). The images were obtained stacking 10 consecutive confocal scans (z step 0.3 µm, total thickness 3 µm) taken in the outer CA1 SP and show two active (OX6-positive, (**f2**)) microglia cells phagocytosing HDN neurons. Scale bar: 10 µm. (**g**) Enlargement of the IBA1-positive (blue) and OX6-positive (red) microglia cells indicated by the white arrows in (**d**). The images were obtained stacking 10 consecutive confocal scans (z step 0.3 µm, total thickness 3 µm) and show rod microglia with a rod “tail” (arrowheads) and a phagocytic “head” (arrows). The neurons were eliminated for clarity. Scale bar: 7.5 µm. (**h**) Representative confocal images of IBA1-positive microglia (green) and DAPI-positive nuclei (open arrows, blue) in CA1 of an OGD organotypic hippocampal slice captured with a 63× objective. The image was obtained by stacking 10 consecutive confocal scans (z step 0.3 µm, total thickness 3 µm). It is clearly visible that 5 rod microglia cells form a train. Scale bar: 15 µm.

It is well known that an OGD insult causes degeneration and morphological alterations of CA1 pyramidal neurons. As previously published [32], we confirm that neurons of the CA1 Stratum pyramidale (SP) of organotypic hippocampal slices showed selective neurotoxicity 24 h after OGD. Indeed, as shown in CA1 SP, we observed numerous pyknotic neurons (Figure 3a) characterized by a very small, highly condensed nucleus (High Density Nucleus Neurons, HDN), typical of apoptotic cells [33]. We had previously shown that NIR laser radiation significantly reduces the increase in HDN neurons induced by OGD, showing a neuroprotective effect [32].

We also found scattered neurons with a karyorrhectic nucleus (Low Density Nucleus neurons, LDN), in CA1 of OGD 24 h, which were too sparse to allow quantitation. We confirmed and extended these findings demonstrating that HDN neurons, although significantly more numerous in the entire CA1 SP of slices subjected to OGD than in control slices, showed a spatial segregation. Indeed, in OGD-treated slices the density of HDN neurons was significantly higher in the outer layer than in the inner layer of CA1 SP (+189%, ** *p* < 0.01 outer layer OGD vs. inner layer OGD, Figure 3b). We also found that the density of HDN neurons was significantly higher in the proximal CA1 SP than in the distal CA1 SP (+56%, * *p* < 0.05 distal CA1 SP OGD vs. proximal CA1 SP OGD, Figure 3c).

**Figure 4:**
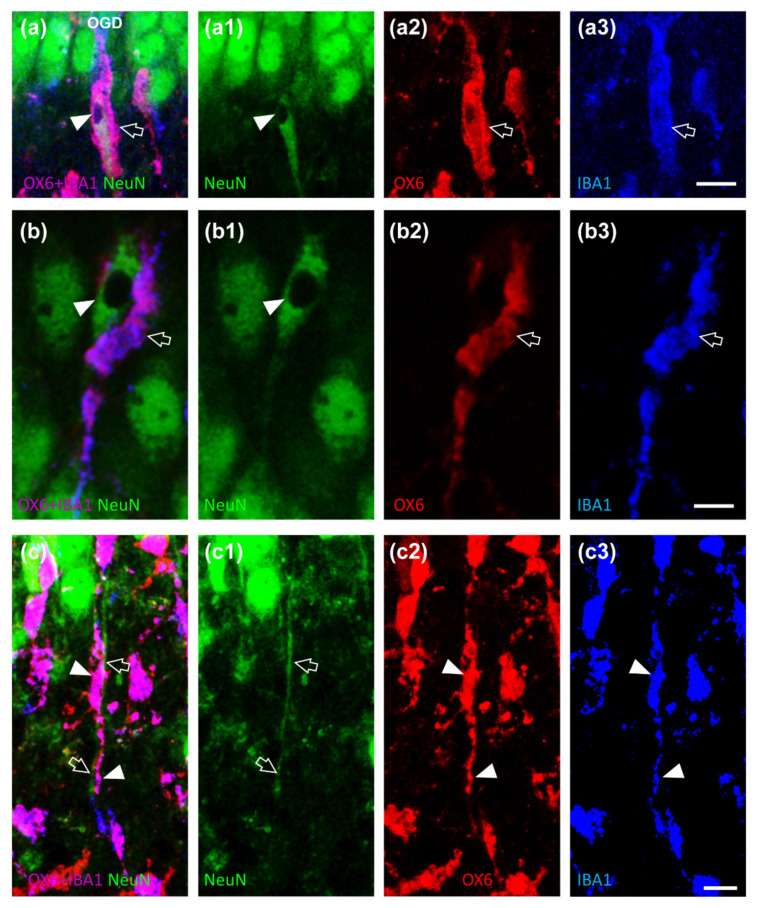
(**a**–**c3**) Representative confocal images of NeuN-positive neurons (green, (**a1**,**b1**,**c1**)), OX6-positive active microglia (red, (**a2**,**b2**,**c2**)) and IBA1-positive total microglia (blue, (**a3**,**b3**,**c3**)) in CA1 SP of OGD organotypic hippocampal slices captured with a 63× objective, stacking 5 consecutive confocal scans (z step 0.3 µm, total thickness 1.5 µm). The merge of the three immunofluorescent staining is shown in (**a**–**c**) in which the pink-purple color is indicative of active (OX6-positive) microglia cells (IBA1-positive). (**a**–**a3**,**b**–**b3**) It is possible to notice that active (OX6-positive, a2, b2, open arrow) microglia cells (IBA1-positive, a3, b3, open arrow) phagocytose an LDN neurons (NeuN-positive, (**a**,**a1**), (**b**,**b1**), arrowhead). Scale bar (**a**–**a3**): 15 µm. Scale bar (**b**–**b3**): 7.5 µm. (**c**–**c3**) It is possible to notice in pink-purple color ((**c**), arrowheads) an active (OX6-positive, (**c2**), arrowheads) microglia cells (IBA1-positive, (**c3**), arrowheads) adjacent to apical dendrites of pyramidal neurons that project in the SR (NeuN-positive, (**c**,**c1**), open arrows).

Triple staining immunohistochemistry for neurons (green), activated microglia (red) and total microglia (blue) showed that most HDN neurons in both the outer and in the inner layers of CA1 were phagocytosed by activated microglia, as shown by the qualitative confocal images (Figure 3d–d3,f–f3) and by the quantitative analysis reported in the graph of Figure 3e. Figure 3d–d3,f–f3 are z projections of five confocal scans acquired with a 63× objective (0.3 µm each, total thickness 1.5 µm in the depth of the organotypic slices). Neurons were immunostained in green (Figure 3d1,f1), activated microglia in red (Figure 3d2,f2), while total microglia were immunostained in blue (Figure 3d3,f3). The pink-purple color (Figure 3d,f, merge of the 3 confocal scans) shows that most microglia cells (blue) were positive for MHCII (red) indicating that they were in an activated state. The open arrows point to pyknotic nuclei of HDN neurons phagocytosed by activated microglia cells. It is interesting to point out that in the trains activated microglia cells (indicated by the white arrows in Figure 3d–d3) acquire a twofold phenotype. An elongated rod-shaped “tail” that spans through the entire thickness of the stratum pyramidalis (arrowheads in Figure 3d2,d3), and a round-shaped amoeboid phagocytic “head” (white arrows in Figure 3d2,d3), which are better visualized in their enlargement (Figure 3g). Figure 3f–f3 also show two activated microglia cells (pink-purple) phagocytosing HDN neurons (green) with their round-shaped amoeboid cell body. We show (Figure 3h) that in the CA1 area of the hippocampus rod microglia align end-to-end one another to form trains, confirming previous findings in other brain regions and different types of insult [14,34]. Indeed, 5 rod microglia cells (evidenced by the presence of DAPI positive nuclei in blue, open arrows, Figure 3h) form a train 175 µm long, that spans from the SR into the SP (not shown). The trains are often adjacent to apical dendrites of pyramidal neurons that project in the SR (see Figure 4c–c3). From the comparison of the quantitative analyses of HDN neurons (Figure 3b) and of phagocytic events of HDN neurons by activated microglia (Figure 3e), it is evident that the phagocytosis takes place mostly in the outer layer of CA1 SP, where most HDN neurons are located. Comparing the data of HDN neurons and phagocytic microglia in the inner and outer layers, we found that about 92% of HDN neurons in the inner layer and 96% of HDN neurons in the outer layer of CA1 SP were phagocytosed by microglia.

Furthermore, some neurons in CA1 SP showed signs of karyorrhexis, as demonstrated by the lack of nuclear staining (LDN neurons, Figure 4a,a1,b,b1, arrowheads). LDN neurons were too sparse throughout the CA1 SP even after OGD to allow quantitation, and no spatial organization within CA1 SP was apparent. Nevertheless, triple immunostaining with NeuN (green), OX6 (red), and IBA1 (blue) demonstrated that many LDN neurons were phagocytosed by activated rod microglia (Figure 4a,b, open arrows) that enveloped the LDN neuronal cell body.

With the triple immunostaining, it was also possible to demonstrate that many rod microglia aligned with apical dendrites of CA1 pyramidal neurons that project into the SR (Figure 4c–c3, open arrows). This spatial localization may help the motility of rod microglia towards the outer layer of CA1, in which most of the damaged neurons are located, in order for the microglia cells to acquire a phagocytic phenotype and eliminate neurons damaged by the OGD insult.

**Figure 5:**
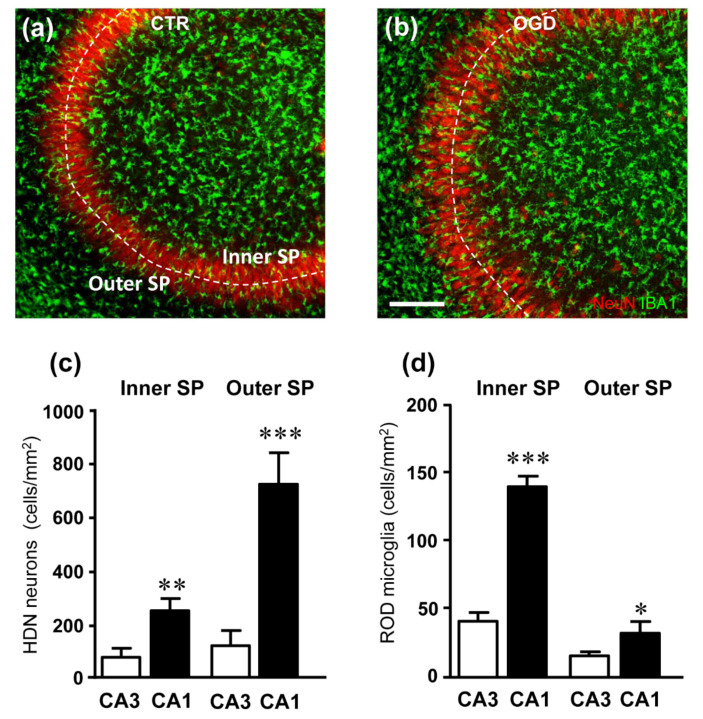
(**a**,**b**) Representative confocal images of NeuN-positive neurons (red) and IBA1-positive total microglia (green) in CA3 hippocampus of a CTR (**a**) and an OGD (**b**) organotypic hippocampal slices captured with a 20× objective. Images were obtained stacking 3 consecutive confocal scans (z step 1.2 µm, total thickness 3.6 µm). It appears that HDN neurons and rod microglia were not significantly more numerous in the OGD slice than in the control slice. The dotted lines delineate the inner and outer CA3 SP layers. Scale bar: 125 µm. (**c**) Quantitative analysis of HDN neurons density (cells/mm^2^) in the inner and outer layers of CA3 and CA1 SP of slices subjected to OGD. HDN neurons were significantly more numerous in both the inner and outer CA1 SP than in the inner and outer CA3 SP, respectively. Statistical analysis: ** *p* < 0.01, inner CA1 SP vs. inner CA3 SP; *** *p* < 0.001, outer CA1 SP vs. outer CA3 SP. (**d**) Quantitative analysis of rod microglia density (cells/mm^2^) in the inner and outer layers of CA3 and CA1 SP of slices subjected to OGD. Rod microglia were significantly more numerous in both the inner and outer CA1 SP than in the inner and outer CA3 SP, respectively. Statistical analysis: *** *p* < 0.001, inner CA1 SP vs. inner CA3 SP; * *p* < 0.05, outer CA1 SP vs. outer CA3 SP. In the bar graphs, each bar represents the mean ± SEM of 7–11 independent slices. All statistical analyses were performed by Student’s paired *t* test.

It is well known that CA1 and CA3 regions of the hippocampus, often considered as a continuum, respond differently to ischemia [35,36]. Therefore, we analyzed both neuronal degeneration and rod-microglia formation in the CA1 and CA3 area of organotypic hippocampal slices, 24 h after OGD (Figure 5). Figure 5a,b show the merged confocal scans of neurons (red) and microglia (green). The quantitative analyses of HDN neurons and rod microglia were performed in the inner and outer layers of the CA3 SP, demonstrating that HDN neuron density in CA1 was significantly higher than in CA3, both in the inner and outer layers (** *p* < 0.01, and *** *p* < 0.001, CA1 vs. CA3 in the inner and outer layer, respectively). Interestingly, the formation of rod microglia was significantly lower in CA3 than in CA1, and it was not different from controls (*** *p* < 0.001, and ** *p* < 0.01, CA1 vs. CA3 in the inner and outer layer, respectively).

**Figure 6:**
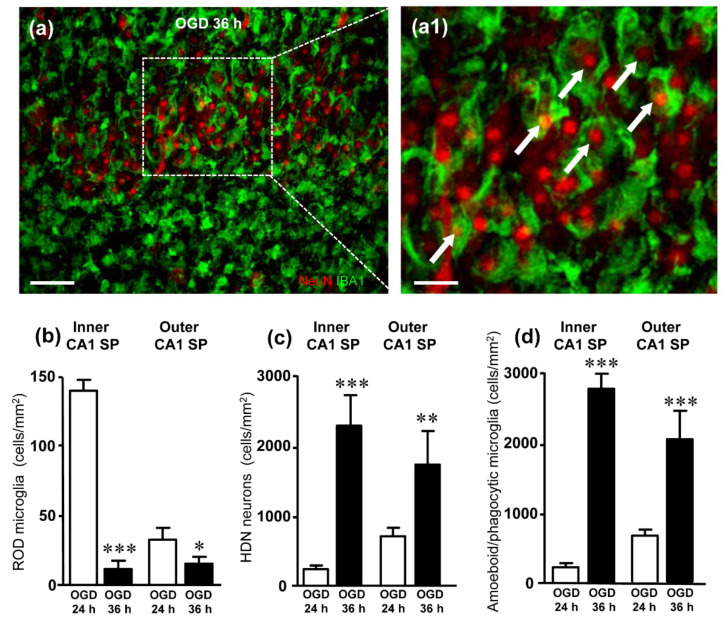
(**a**) Representative confocal images of NeuN-positive neurons (red) and IBA1-positive microglia (green) in CA1 hippocampus of an OGD organotypic hippocampal slices, harvested 36 h after the insult. The images, captured with a 40× objective, were obtained stacking 5 consecutive confocal scans (z step 0.5 µm, total thickness 2.5 µm). (**a1**) Enlargement of the framed area in A. In CA1 SP, most microglia cells had acquired an amoeboid phagocytic conformation and numerous HDN neurons were phagocytosed by microglia (arrows). (**b**) Quantitative analysis of rod microglia density (cells/mm^2^) in the inner and outer CA1 SP of OGD 24 h and OGD 36 h slices. Rod microglia were significantly less numerous in both the inner and outer CA1 SP 36 h after OGD than 24 h after OGD. Statistical analysis: *** *p* < 0.001, OGD 36 h vs. OGD 24 h; * *p* < 0.05, OGD 36 h vs. OGD 24 h. In the bar graphs, each bar represents the mean ± SEM of 6–8 experiments. All statistical analyses were performed by Student’s paired *t* test. (**c**) Quantitative analysis of HDN neurons density (cells/mm^2^) in the inner and outer CA1 SP of OGD 24 h and OGD 36 h slices. HDN neurons were significantly more numerous in both the inner and outer CA1 SP 36 h after OGD than 24 h after OGD. Statistical analysis: *** *p* < 0.001, OGD 36 h vs. OGD 24 h; ** *p* < 0.001, OGD 36 h vs. OGD 24 h. (**d**) Quantitative analyses of the density of amoeboid/phagocytic microglia (cells/mm^2^) in the inner and outer layers of CA1 SP. Statistical analysis: *** *p* < 0.001, OGD 36 h vs. OGD 24 h. In the bar graphs, each bar represents the mean ± SEM of 4–9 independent slices. All statistical analyses were performed by Student’s paired *t* test.

Finally, we analyzed neurodegeneration and rod microglia at 36 h after the ischemic insult in CA1 (Figure 6). Interestingly, 36 h after OGD the density of rod microglia was significantly lower than in the corresponding layers of CA1 24 h after OGD (Figure 6b). At this time point after the insult, neurodegeneration was more intense than at 24 h, as shown by the significantly higher density of HDN neurons localized in both the inner and outer layers of CA1 (Figure 6c). Moreover, most microglia cells had acquired an amoeboid, phagocytic conformation (Figure 6a1), and quantitative analyses show that the density of amoeboid cells at 36 h was significantly higher than at 24 h in both CA1 inner and outer layers (Figure 6d). From the comparison of the quantitative analyses of HDN neurons (Figure 6c) and of amoeboid/phagocytic microglia (Figure 6d), it is evident that phagocytosis takes place to a similar extent in both layers of CA1 SP. Comparing the data of HDN neurons and phagocytic microglia in the inner and outer layers, we found that about 82% of HDN neurons in the inner layer and 84% of HDN neurons in the outer layer of CA1 SP were phagocytosed by microglia.

## 3. Discussion

**Figure 7:**
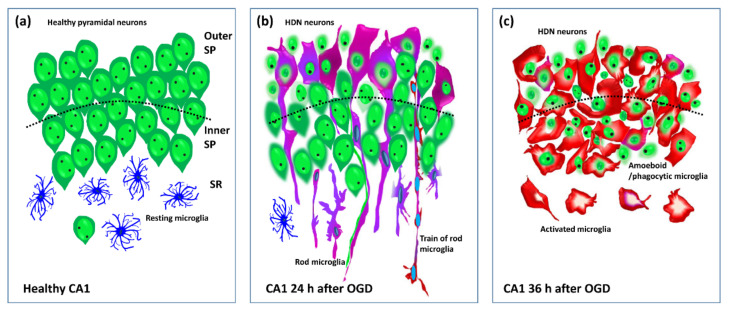
Schematic representation of the healthy CA1 hippocampus (**a**) and the morphofunctional modifications of microglia and neurons at 24 (**b**) and 36 h (**c**) after the OGD. Resting microglia (blue), activated rod microglia (pink-purple), amoeboid/phagocytic microglia (red). A train of rod microglia is shown (**b**).

In this paper, we demonstrated that an OGD insult induced rapid, time- and space-dependent phenotypic modifications of microglia in both the CA1 SP and SR of rat organotypic hippocampal slices. As expected, the simil-ischemic insult caused the degeneration of numerous pyramidal neurons which showed a spatial distribution, being mainly localized in the proximal part and in the outer layer of the CA1 SP 24 h after the insult. In addition, at 24 h after the insult, a significant percentage of microglia acquired a rod phenotype. Rod microglia formed trains of elongated cells, which spanned from the SR throughout the entire thickness of CA1, to reach the outer layer of SP where they acquired a round-shaped amoeboid phagocytic head. The majority of the damaged pyknotic HDN neurons located in the outer layer of CA1 SP were phagocytosed by amoeboid microglia. NIR-laser application, a treatment known to preserve neuronal viability 24 h after OGD [32], prevented the formation of rod microglia in both SP and SR of CA1. In CA3 SP, an area known to be more resistant to ischemia [35], the scenario was completely different. At 24 h after OGD, pyramidal neurons were significantly less damaged, and, in parallel, microglia did not show rod-like morphological modifications. We followed the time-course of the morphofunctional changes of microglia caused by OGD in CA1. At 36 h after the OGD, the neuronal damage was significantly more pronounced in the entire CA1 SP, while rod microglia cells were no longer detectable in any of the CA1 areas investigated. At this time after the insult, most of microglia had acquired an amoeboid, phagocytic morphology. HDN, pyknotic neurons were significantly more numerous at 36 than at 24 h after OGD, and were located throughout the SP, most of which were phagocytosed by amoeboid microglia in both inner and outer layers of CA1 SP. A schematic representation of OGD-induced microglia and neuron modifications in CA1 is shown in Figure 7.

To our knowledge, this is the first demonstration that microglia respond to an ischemic insult by acquiring the rod phenotype in the CA1 hippocampus. Although rod microglia morphology suggests specific functions in pathological states, very little is known on their role in ischemia or other neurological pathologies.

At 24 h after OGD, microglia cells had elongated their processes from their apical and basal ends, acquiring a rod shape. Rod microglia expressed MHCII, a known marker for activated microglia, as evidenced by their OX6 immunopositivity. We found that rod microglia cells formed trains in which rod cells were linked end-to-end. The trains, which spanned at least 175 µm, were formed in the CA1 SR, were oriented with their longest axis perpendicular to the CA1 SP and were aligned parallel and in close contact with the apical dendrites of CA1 pyramidal neurons, perpendicular to the CA1 main axis. A similar spatial disposition, with rod cells and trains perpendicular to the pial surface of the brain, had been shown in animal models of TBI [14] and in human brain pathologies, such as viral encephalitis, HIV-1 and general paralysis of the insane [37]. Furthermore, the trains of rod microglia closely recall those aligned with injured axons first detected by Nissl [25,26,38,39].

How trains of microglia are formed, and their functional role has still not been understood. Several hypotheses have been proposed. We believe that the alignment of rod microglia with apical dendrites of CA1 pyramidal neurons may help tracking microglia towards the areas with higher injury. Trains of rod microglia may help migration of microglia from the SR towards the SP, to infiltrate and cross it to reach the outer layer of SP to find and phagocytose damaged neurons [40]. Indeed, it has been previously shown that rod microglia migrate in response to chemokine signaling that originates from damaged neurons or other cells [4,41]. Other hypotheses [15] are that rod microglia form trains adjacent to neurons (i) to splint damaged neuronal processes, (ii) to form a barrier to protect uninjured neurons from a damaging environment, (iii) to seal a damaged neuron from further interaction with healthy neighboring neurons. Nevertheless, the question is still open.

Most of the rod microglia located in the outer layer of CA1 SP, along with an elongated tail, had an enlarged head that phagocytosed damaged, pyknotic neurons. These findings are in agreement with those of Ziebell and collaborators [14] who showed that rod microglia exhibit high immunoreactivity for the phagocytic marker ED1, confirming that rod microglia are highly phagocytic cells [15,38,42,43]. It has been demonstrated in the spinal cord zebrafish that microglia constantly move their elongated processes to patrol the parenchyma [23]. In this way, in response to damaging stimuli, a quick activation of microglia leads to morphological and functional changes that promote the elongation of the cells that migrate to the areas affected by damage [3,22,23] where they acquire the amoeboid phagocytic form and eliminate the damaged neurons by phagocytosis. If the tissue is not completely damaged, phagocytic microglia, after fulfilling their functions, are able to return to their resting state and continue patrolling the tissue [23]. On the contrary, we demonstrated here that in a tissue that is extensively damaged after OGD, the time course of microglia activation proceeds from rod microglia to amoeboid, phagocytic microglia in 12 h.

Accumulating evidence suggest that rod microglia originate from resident CNS microglia [19,28,44] by the differentiation or proliferation of existing microglia. In accordance with these previous findings, our data confirm that rod microglia morphologically differentiate from existing microglia, since we demonstrated that no differences in the number of IBA1 positive microglia was found among the experimental groups.

The time course of rod microglia emergence and resolution in most diseases is poorly understood since clinical cases are usually based on a single post-mortem histological observation. Nevertheless, in a rat model of transient ischemic attack obtained experimentally, with the middle cerebral artery occlusion, rod microglia were found in the penumbra area of the ipsilateral cortex already 10 min after the insult, and at 24 or 48 h of reperfusion [45]. On the contrary, in our experiments rod microglia was no longer evident in CA1 SP or SR 36 h after OGD, when frank neuronal damage and degeneration of CA1 SP were evident. Our data can be explained considering that the 30 min OGD is a very strong insult and confirm the findings by Graeber and Mehraein [37] who determined that rod microglia can only form when the tissue is preserved and not completely damaged after the insult. Indeed, 36 h after OGD, when most of CA1 pyramidal neurons were damaged, the density of rod microglia was very low, while microglia cells were mainly amoeboid and were phagocytosing HDN neurons in both the outer and inner SP layers. Our data indicate that in 12 h the scenario completely changed, and most of microglia assumed an amoeboid phagocytic phenotype. These results are in accordance with Graeber and Mehraein [37], who described that when the tissue is highly damaged and with extensive neuronal necrosis, microglia acquire the morphology of activated, phagocytic cells. Until recently, activated microglia was classified in M1, proinflammatory, and M2, anti-inflammatory, states [46]. Nevertheless, this classification is too simplistic and does not correspond to the many different microglia phenotypes found in the brain [47,48,49,50]. Indeed, it is now more and more evident that many microglia activation states may exist, not related merely to the type of insult and the time course of the disease progression, but dependent upon the CNS area in which microglia cells are located [47,51,52,53,54,55,56,57]. In this scenario, it is not surprising that microglia located in two contiguous hippocampal areas, CA1 and CA3, react in a different way to the same simil-ischemic insult, responding to internal cues, independent from the environment or from the insult. The areas CA3 and CA1 of the hippocampus are considered as a continuum since they are contiguous and interconnected via the Schaffer collaterals. Nevertheless, they respond differently to ischemic insults. The CA1 pyramidal neurons are among the most vulnerable [35], as demonstrated in experimental animal models of hypoxia or ischemia (reviewed by [58,59,60]), and in patients [61,62,63,64]. Although it is still not completely understood how and why these two areas of the hippocampus are differently affected by the same ischemic event, some new data start to shed some light onto the possible mechanisms. The modifications that ischemia brings about not only in neurons but also in microglia, or astrocytes, and their interrelationships can be envisaged as one of the causes of the different responses of CA1 and CA3 neurons to the same ischemic insult. Our data may help to better understand these different responses. We found that, at 24 h after OGD, higher neuronal vulnerability was paralleled by rod microglia formation in CA1. On the contrary, in CA3 where most of pyramidal neurons did not show signs of damage, in parallel no rod microglia was formed. Furthermore, after NIR-laser application, a physical stimulus known to protect neurons after OGD [32], no rod microglia were found in CA1. Although from our data it is not possible to discriminate which came first, the presence of neuronal damage was always accompanied by rod microglia formation. From all our results it is possible to envisage that rod microglia do not have a protective role towards neuronal damage caused by an ischemic insult, at least in CA1 hippocampus.

Area CA2 of the hippocampus, located between CA1 and CA3 [65] is beginning to emerge as an interesting hippocampal area with important functions, and CA2 neurons seem to be relatively resistant to ischemic insult [66,67,68,69,70,71]. It would, therefore, be very interesting to verify the morphofunctional modifications of microglia in the CA2 area after OGD.

## 4. Materials and Methods

### 4.1. Animals

The experimental procedures were conducted in accordance with the ARRIVE guidelines and were authorized by the Italian Ministry of Health. The ethical policy of the University of Florence complies with to the Directive 2010/63/EU of the European Parliament and to the Italian Regulation DL 26/2014 on the protection of animals used for scientific purposes. According to the law, all efforts were made to fulfill to the principle of 3Rs. The authors further attest that all efforts were made to minimize the number of animals used and their suffering.

### 4.2. Preparation of Rat Organotypic Hippocampal Slice Cultures

Male and female Wistar rat pups (7–9 days old) were obtained from Charles River (Milano, Italy). Animals were housed at 23 ± 1 °C under a 12 h light–dark cycle (lights on at 07:00) and were fed a standard laboratory diet with ad libitum access to water.

Organotypic hippocampal slice cultures were prepared as previously reported [72,73]. Briefly, hippocampi were removed from the brains of 7- to 9-day old Wistar rat pups (Harlan, MI, Italy) and transverse slices (420 µm) were prepared using a McIlwain tissue chopper. Before experiments, all slices were screened for viability by incubating them for 30 min with propidium iodide (PI, 5 μg/mL); slices displaying signs of neurodegeneration were discarded from the study.

Slices were then transferred onto 30 mm diameter semiporous membranes inserts (Millicell CM PICM03050; Millipore, Italy), which were placed in six well tissue culture plates containing 1.2 mL medium per well. Slices were maintained at 37 °C in an incubator in atmosphere of humidified air and 5% CO_2_ for 14 days.

### 4.3. OGD Exposure in Rat Organotypic Hippocampal Slices

Organotypic hippocampal slices were exposed to OGD as previously detailed [74,75], while others were not exposed to OGD treatment (CTR). Briefly, OGD was reproduced by exposing the slices to serum- and glucose-free medium saturated with 95% N_2_ and 5% CO_2_ for 30 min at 37 °C in an airtight anoxic chamber equipped with an oxygen gas controller (BioSpherix, New York, NY, USA). The cultures were then transferred to oxygenated serum-free medium (75% Eagle’s minimal essential medium; 25% Hank’s balanced salt solution; 2 mM L-glutamine; and 3.75 μg/mL amphotericin B) containing 5 mg/mL glucose and returned to the incubator under normoxic conditions. Slices were harvested 24 h (OGD) or 36 h after the insult (OGD 36 h). A group of organotypic hippocampal slices were not exposed to OGD treatment (CTR).

### 4.4. NIR-Laser Treatment in Organotypic Hippocampal Slices

NIR laser treatment was performed as previously described [32]. Briefly, hippocampal slice cultures were treated with NIR laser (exposure time 12 s, fluence 7.42 J/cm^2^), alone (Laser) or delivered immediately after OGD insult (OGD+Laser). Slices were harvested 24 h after the insult. NIR-Laser treatment was performed with a Multiwave Locked System laser (MLS-MiS, ASA S.r.l., Vicenza, Italy), a class IV NIR laser with two synchronized sources (laser diodes). The first of these is a pulsed laser diode emitting at 905 nm wavelength, with peak power from 140 W ± 20% to 1 kW ± 20% and pulse frequency varying in the range 1–2000 Hz; the second laser diode emitting at 808 nm wavelength can operate in continuous (max power 6 W ± 20%) or frequenced (repetition rate 1–2000 Hz, 50% duty cycle) mode. The two laser beams work simultaneously, synchronously and the propagation axes are coincident.

### 4.5. Fluorescence Immunohistochemistry

At the end of the experiments, organotypic hippocampal slices were harvested and fixed overnight in ice-cold paraformaldehyde (4% in PBS). The day after, slices were washed in PBS and placed for at least 48 h in a sucrose solution (18% in PBS). Triple labelling fluorescent immunohistochemistry was performed with the free-floating method [75,76]. Day 1, Organotypic hippocampal slices were placed in a multiwell and blocked for 60 min with BB containing 10% Normal Goat Serum. All antibodies were diluted in BB. Slices were then incubated overnight at 4 °C under slight agitation with a combination of two different primary antibodies: a mouse anti-OX6 antibody (1:200; Code #554926, BD Pharmingen, San Diego, CA, USA) for activated microglia and a rabbit anti-IBA1 antibody (1:300; Code #016-20001, WAKO, Osaka, Japan) for total microglia.

Day 2, slices were incubated for 2 h at room temperature in the dark with AlexaFluor 635 goat anti-rabbit IgG (1:400; Code #A31577, Thermo Fisher Scientific, Waltham, MA, USA) secondary antibody. Slices were then incubated for 2 h at room temperature in the dark with AlexaFluor 555 donkey anti mouse IgG (1:400; Code #A31570, Thermo Fisher Scientific) plus AlexaFluor 635 goat anti-rabbit IgG (1:400; Code #A31577, Thermo Fisher Scientific). Neurons were immunostained using a mouse anti-NeuN antibody conjugated with the fluorochrome AlexaFluor 488, for 2 h at room temperature in the dark (1:100 Code #MAB377X, Millipore). Finally, slices were mounted onto gelatin-coated slides using Vectashield mounting medium with DAPI (Code #H-1200, Vectashield, Burlingame, CA, USA).

### 4.6. Microscopy Techniques and Quantitative Analysis

Confocal microscopy acquisitions were performed in CA1 dorsal hippocampus to acquire immunofluorescence signals of NeuN, IBA1 and OX6 and DAPI fluorescence. Slices were observed under a LEICA TCS SP7 confocal laser scanning microscope (Leica Microsystems CMS GmbH) equipped with a 20× and a 63× objectives. The parameters of acquisition were maintained constant, with a frame dimension 1024 × 1024 pixels, frequency of acquisition 200 Hz, z step of 1.2 μm for 20× acquisition and 0.3 μm for 63× acquisition. Quantitative analyses were made using ImageJ software (National Institute of Health, http://rsb.info.nih.gov/ij, accessed on 30 July 2021). We focused our count on three different regions of interest (ROIs). The ROIs areas were calculated in mm^2^, with ImageJ software:

− Inner CA1 Stratum Pyramidalis (SP), determined as the 50% of total CA1 area located toward the Stratum Radiatum (SR);
− Outer CA1 SP, determined as the 50% of total CA1 area located toward the Stratum Oriens.
− CA1 SR;
− Proximal CA1 SP, determined along the transverse axis, as the section of CA1 bordering CA2 [66].
− Distal CA1 SP, determined along the transverse axis as the section of CA1 bordering subiculum [66].

Rod microglia density: We counted rod microglia and the total microglia on z-projection of three consecutive confocal planes (total thickness 3.6 µm), separately. Rod microglia and total microglia densities were expressed as cells/mm^2^. Rod microglia was also expressed as percent of rod microglia on total microglia.

HDN neurons density: HDN neurons are pyknotic neurons with nuclei that have a highly condensed NeuN-positive nucleus and very faint NeuN-positive cytoplasmic labeling [32,77,78]. To be able to identify separately each neuron in the dense CA1 pyramidal cell layer, we counted HDN neurons in a single confocal scan (thickness 1.2 µm) taken in the depth of the slice. HDN-neuron density in inner and outer CA1 density was expressed as HDN neurons/mm^2^, separately.

Density of activated microglia phagocytosing HDN neurons. Thanks to triple labelling fluorescent immunohistochemistry (NeuN, IBA1 and OX6), it was possible to distinguish and quantify activated microglia (IBA1-positive and OX6-positive) while phagocytosing NeuN-positive HDN neurons. We counted phagocytic microglia on a single confocal plane (thickness 1.2 µm) in the depth of the slice. Density of phagocytic microglia was expressed as HDN phagocytic microglia/mm^2^ in inner and outer CA1, separately.

### 4.7. Statistical Analysis

Raw data are provided in the Supplementary Material. In the figures, data are presented as means ± SEM of n experiments from independent organotypic slices. Statistical analyses were performed by One-way ANOVA followed by Newman-Keuls post hoc test or Student’s paired *t* test as appropriate. All statistical analyses were performed using Graph-Pad Prism v. 5 for Windows (GraphPad Software, San Diego, CA, USA). A probability value *p* < 0.05 was considered significant.

## 5. Conclusions

All these data taken together demonstrate that, in the hippocampus, in response to a damaging stimulus, microglia can acquire different morphofunctional phenotypes which depend not only on the time after the insult but also on the hippocampal subregion in which the microglia are located.

## Data Availability

Our own data presented in this study are available on request from the corresponding author.

## Supplementary Materials

The following supporting information can be downloaded at: https://www.mdpi.com/article/10.3390/ijms23031422/s1.
