# Supplementary material for: Hypoxia/Ischemia-Induced Rod Microglia Phenotype in CA1 Hippocampal Slices
Source: Int J Mol Sci. 2022 Jan 26;23(3):1422. doi: 10.3390/ijms23031422 (PMC8836225; doi:10.3390/ijms23031422)
Supplement: Supplementary file 1 [file ijms-23-01422-s001.zip › ijms-1542505-supplementary.pdf]

Supplementary Material

Raw Data.

Density ROD microglia in Inner CA1 SP (cells/mm²)			
CTR	OGD	OGD+LASER	LASER
33,37477	125,7208	66,06765	65,03159
20,08597	159,9488	71,9321	12,64111
30,18169	150,2584	88,98029	98,0216
41,52565	109,6638	32,64666	40,03737
21,48182	137,5686	36,36231	23,24203
37,44243	127,2462	25,76257	47,99846
	167,7121		
30,68 ± 3,501	139,7 ± 7,812	53,63 ± 10,42	47,83 ± 12,52
n=6	n=7	n=6	n=6

Density ROD microglia in Outer CA1 SP (cells/mm²)			
CTR	OGD	OGD+LASER	LASER
32,47649	26,81612	27,16985	9,204967
14,55329	9,426847	18,41112	12,65999
30,21574	22,18894	19,46321	97,09209
42,44843	60,50339	18,171	13,35773
43,82985	14,86083	21,67959	22,66443
49,32487	24,54771	34,35128	32,1916
	70,35194		
35,47 ± 5,114	32,67 ± 8,813	23,21 ± 2,612	31,20 ± 13,62
n=6	n=7	n=6	n=6

Density ROD microglia in CA1 SR (cells/mm²)			
CTR	OGD	OGD+LASER	LASER
8,184378	82,87407	97,29204	17,65303
17,02388	127,6305	43,42405	19,87637
42,27913	95,50585	31,4599	17,98189
34,92189	57,30057	49,96669	21,16537
22,94736	84,70485	15,78881	26,64062
21,19744	129,9699	35,52082	
	88,88988		
24,43 ± 5,034	95,27 ± 9,758	45,58 ± 11,38	20,66 ± 1,626
n=6	n=7	n=6	n=5

Percent ROD/TOT microglia in Inner CA1 SP			
CTR	OGD	OGD+LASER	LASER
5,333333	17,64706	6,944445	10,44776
4,109589	29,82456	6,896552	2
4,347826	25,42373	15,25424	9,523809
5,128205	17,64706	4,705883	4,411765
2,325581	16,12903	5	2,631579
5,555555	17,64706	4,225352	6,122449
	23,25581		
4,467 ± 0,4865	21,08 ± 1,951	7,171 ± 1,683	5,856 ± 1,437
n=6	n=7	n=6	n=6

Percent ROD/TOT microglia in Outer CA1 SP			
CTR	OGD	OGD+LASER	LASER
5,479452	3,614458	3,571429	1,408451
2,380952	1,111111	0,8264463	2
4,6875	2,898551	1,923077	1,886792
5,263158	8,928572	2,941176	3,636364
6,666667	1,666667	1,149425	3,703704
8,888889	2,857143	2,803738	
	8,695652	5,128205	
5,561 ± 0,881	4,253 ± 1,218	2,621 ± 0,561	2,527 ± 0,477
n=6	n=7	n=7	n=5

Percent ROD/TOT microglia in CA1 SR			
CTR	OGD	OGD+LASER	LASER
1,515152	14,11765	16,85393	2,702703
2,380952	23,28767	13,18681	2,816901
7,142857	18,60465	7,608696	2,597403
5	13,33333	4,878049	2,564103
2,666667	10,75269	6,741573	1,333333
3,030303	17,74194	1,923077	3,846154
	13,11475	4,395605	
3,623 ± 0,847	15,85 ± 1,613	7,941 ± 1,992	2,643 ± 0,327
n=6	n=7	n=7	n=6

Density TOT microglia in Inner CA1 SP (cells/mm²)			
CTR	OGD	OGD+LASER	LASER
625,777	712,4178	951,3742	622,4452
488,7585	536,299	737,4971	632,0553
694,179	591,0165	1043,015	1029,227
809,7502	621,4283	583,3152	907,5137
923,7181	852,9254	693,7416	883,1971
673,9638	721,0621	727,2463	783,9749
	721,1619	609,7142	
702,7 ± 61,34	679,5 ± 39,74	763,7 ± 64,81	809,7 ± 65,94
n=6	n=7	n=7	n=6

Density TOT microglia in Outer CA1 SP (cells/mm²)			
CTR	OGD	OGD+LASER	LASER
592,696	741,9127	760,7559	653,5527
611,238	848,4163	817,3356	632,9995
644,6025	765,5184	957,3782	695,8267
806,5201	677,6379	661,749	707,9599
657,4478	891,6497	710,8773	623,2719
554,9047	859,1698	773,2387	869,1733
	809,0472	669,8499	
644,6 ± 35,70	799,1 ± 28,39	764,5 ± 38,57	697,1 ± 37,05
n=6	n=7	n=7	n=6

Density TOT microglia in CA1 SR (cells/mm²)			
CTR	OGD	OGD+LASER	LASER
540,1689	587,0247	727,7723	653,1621
715,0032	548,0604	737,798	705,6111
591,9078	513,3439	570,7161	692,3029
698,4378	429,7543	644,9279	825,4492
860,5259	787,7551	741,1725	728,2755
699,5156	732,5574	821,0181	692,6561
	677,7853	808,0988	
684,3 ± 45,37	610,9 ± 48,16	721,6 ± 33,34	716,2 ± 24,01
n=6	n=7	n=7	n=6

Density HDN neurons CA1 SP (cells/mm²)			
Inner CA1 SP		Outer CA1 SP	
CTR	OGD	CTR	OGD
28,6723	167,8259	25,2553	786,986
34,0567	207,4904	30,7319	845,1881
30,0908	358,1209	29,8547	1291,634
29,9990	365,0489	22,1245	431,0103
28,7576	449,3525	19,8956	1010,708
21,1222	107,7194	21,1852	430,1017
27,2121	299,2131	20,6987	527,5732
	24,31168		181,2952
	235,3403		902,2675
28,56 ± 1,479	246,0 ± 45,17	24,25 ± 1,690	711,9 ± 115,4
n=7	n=9	n=7	n=9

Density of Amoeboid/Phagocytic microglia in CA1 SP (cells/mm²)			
Inner CA1 SP		Outer CA1 SP	
CTR	OGD	CTR	OGD
22,2312	65,67651	15,3654	283,4856
36,8971	75,1082	22,3489	586,5215
24,5587	189,0756	22,5412	560,3781
25,3321	173,5537	25,5423	721,1259
27,5761	280,8528	27,4762	1072,946
22,3698	262,1782	22,9811	902,5077
28,3415	529,9173	21,3587	755,781
			575,8635
26,76 ± 1,906	225,2 ± 59,61	22,52 ± 1,435	682,3 ± 84,77
n=7	n=7	n=7	n=8

Density HDN neurons in OGD in CA3/CA1 SP (cells/mm²)			
Inner SP		Outer SP	
CA3	CA1	CA3	CA1
323,5747	167,8259	570,1078	786,986
0	207,4904	0	845,1881
114,2117	358,1209	176,5874	1291,634
49,32608	365,0489	52,44939	431,0103
184,6682	449,3525	158,0528	1010,708
34,61206	107,7194	65,40294	430,1017
0	299,2131	0	527,5732
0	24,31168	0	181,2952
0	235,3403	0	902,2675
48,08155		159,2712	
75,45 ± 33,48	246,0 ± 45,17	118,2 ± 55,17	711,9 ± 115,4
n=10	n=9	n=10	n=9

Density ROD microglia in OGD in CA3/CA1 SP (cells/mm²)			
Inner SP		Outer SP	
CA3	CA1	CA3	CA1
78,01408	125,7208	31,20563	26,81612
20,28398	159,9488	17,82325	9,426847
40,53539	150,2584	13,35907	22,18894
22,94499	109,6638	0	60,50339
63,38903	137,5686	25,24679	14,86083
22,62443	127,2462	22,55249	24,54771
45,58467	167,7121	21,89262	70,35194
55,02517		17,53878	
60,03122		11,24568	
8,607704		8,897351	
33,29375		0	
40,94 ± 6,504	139,7 ± 7,812	15,43 ± 2,998	32,67 ± 8,813
n=11	n=7	n=11	n=7

Density ROD microglia in OGD 24h/36h (cells/mm²)			
Inner CA1 SP		Outer CA1 SP	
OGD 24h	OGD 36h	OGD 24h	OGD 36h
125,7208	19,7593	26,8161	9,8459
159,9488	15,5618	9,4268	25,4139
150,2584	0,0000	22,1889	11,5607
109,6638	12,0891	60,5034	16,0522
137,5686		14,8608	
127,2462		24,5477	
167,7121		70,35194	
139,7 ± 7,812	11,85 ± 4,251	32,67 ± 8,813	15,72 ± 3,487
n=7	n=4	n=7	n=4

Density HDN neurons in OGD 24h/36h (cells/mm²)			
Inner CA1 SP		Outer CA1 SP	
OGD 24h	OGD 36h	OGD 24h	OGD 36h
167,8259	3138,0540	786,986	2652,2150
207,4904	1723,1300	845,1881	1188,1440
358,1209	2013,0470	1291,634	1358,7580
365,0489	2305,0364	431,0103	1802,3400
449,3525		1010,708	
107,7194		430,1017	
299,2131		527,5732	
24,31168		181,2952	
235,3403		902,2675	
246,0 ± 45,17	2295 ± 305,1	711,9 ± 115,4	1750 ± 327,3
n=9	n=4	n=9	n=4

Density of Amoeboid/Phagocytic microglia in OGD 24h/36h (cells/mm²)			
Inner CA1 SP		Outer CA1 SP	
OGD 24h	OGD 36h	OGD 24h	OGD 36h
65,67651	3392,7660	283,4856	2834,1360
75,1082	2532,2520	586,5215	1434,3360
189,0756	2436,8460	560,3781	1929,6640
173,5537	2805,0355	721,1259	2100,3260
280,8528		1072,946	
262,1782		902,5077	
529,9173		755,781	
		575,8635	
225,2 ± 59,61	2792 ± 215,00	682,3 ± 84,77	2075 ± 289,9
n=7	n=4	n=8	n=4
